# Recognition of Attentional States in VR Environment: An fNIRS Study

**DOI:** 10.3390/s22093133

**Published:** 2022-04-20

**Authors:** Dariusz Zapała, Paweł Augustynowicz, Mikhail Tokovarov

**Affiliations:** 1 Department of Experimental Psychology, The John Paul II Catholic University of Lublin, 20-950 Lublin, Poland; augustynowicz@kul.pl; 2 Cortivision sp. z o.o., 20-803 Lublin, Poland; 3 Institute of Computer Science, Faculty of Electrical Engineering and Computer Science, Lublin University of Technology, 20-618 Lublin, Poland; m.tokovarov@pollub.pl

**Keywords:** fNIRS, BCI, virtual reality, n-back, head-mounted display, DLPFC, MFG

## Abstract

An improvement in ecological validity is one of the significant challenges for 21st-century neuroscience. At the same time, the study of neurocognitive processes in real-life situations requires good control of all variables relevant to the results. One possible solution that combines the capability of creating realistic experimental scenarios with adequate control of the test environment is virtual reality. Our goal was to develop an integrative research workspace involving a CW-fNIRS and head-mounted-display (HMD) technology dedicated to offline and online cognitive experiments. We designed an experimental study in a repeated-measures model on a group of BCI-naïve participants to verify our assumptions. The procedure included a 3D environment-adapted variant of the classic n-back task (2-back version). Tasks were divided into offline (calibration) and online (feedback) sessions. In both sessions, the signal was recorded during the cognitive task for within-group comparisons of changes in oxy-Hb concentration in the regions of interest (the dorsolateral prefrontal cortex-DLPFC and middle frontal gyrus-MFG). In the online session, the recorded signal changes were translated into real-time feedback. We hypothesized that it would be possible to obtain significantly higher than the level-of-chance threshold classification accuracy for the enhanced attention engagement (2-back task) vs. relaxed state in both conditions. Additionally, we measured participants′ subjective experiences of the BCI control in terms of satisfaction. Our results confirmed hypotheses regarding the offline condition. In accordance with the hypotheses, combining fNIRS and HMD technologies enables the effective transfer of experimental cognitive procedures to a controlled VR environment. This opens the new possibility of creating more ecologically valid studies and training procedures.

## 1. Introduction

Functional near-infrared spectroscopy (fNIRS) is one of the newest and fastest-growing functional neuroimaging techniques. The fNIRS uses infrared light (650–950 nm) to measure the hemodynamic response of the neocortical brain regions [1]. There are several variations of the fNIRS method used in neuroscience research, such as time-domain (TD-fNIRS), or frequency-domain (FD-fNIRS) methods. However, the most common commercially available systems use a continuous wave (CW) NIR light emitted by a laser or LED [2]. This method uses a modified Beer–Lambert law to estimate changes in the concentration of oxy-hemoglobin (HbO) and deoxy-hemoglobin (HbR) based on measuring the difference in the ratio of the injected to the output light after passing through the tissue [3].

The CW fNIRS is appreciated for its: non-invasiveness, relatively high resistance to motion artifacts, higher spatial resolution than electroencephalography (EEG), better temporal resolution than functional magnetic resonance imaging (fMRI), and easy integration with other measurement devices [4] For these reasons, fNIRS is gaining popularity as a research tool in studies on children [5], neurofeedback [6], neurorehabilitation [7], sport [8], brain-computer interfaces [9] and social neuroscience [10]. On the other hand, fNIRS has a worse signal-to-noise ratio and spatial resolution than fMRI [11], limiting its application in the medical field.

As a result of these capabilities and limitations, CW-fNIRS remains one of the few brain-recording methods that are feasible for use in motion and outside the laboratory [12]. These features are particularly relevant to research approaches such as neuroergonomics [13], real-life neuroscience [4], and UX/UI studies [14]. At the same time, the fNIRS developers create more devices that are fully mobile, wireless [15], and designed to work with head-mounted displays (HMD) [16] or training apps [17]. VR technology has been used in the research and training of attentional skills due to its ability to isolate the subject from external distractions [18]. For example, ref. [19] showed that users′ workloads could be detected from the fNIRS signal during the n-back task implemented into the VR scene. The highest classification accuracy (from 62% in person-dependent analysis to 66% in person-adaptive analysis) was obtained for the binary discrimination between 1-back vs. 2-back task performance. However, there were significant individual differences in the results. In other studies, Luong et al. [20] proved that complex VR cognitive training can modulate users′ mental workloads by customizing scenarios to individual cognitive states.

Moreover, Hudak et al. [21] have created fNIRS-based neurofeedback training in a VR environment to reduce impulsive behavior in a group with ADHD. After eight training sessions, a significant reduction in commission errors on the no-go task and a decrease in reaction time variability on the stop-signal tasks for the experimental group was achieved; at the same time, an increase in prefrontal oxygenated hemoglobin concentration was observed in the same group. In a study by Salski et al. [22], an experiment was conducted on a group of children with ADHD comparing the effectiveness of hemoencephalographic-based neurofeedback (HEG-NF) training in three visual modes (standard 2D HEG-NF, simple VR HEG-NF, complex VR HEG-NF). The results showed that children in the VR HEG-NF have better results in cognitive assessment after training than children with standard 2D HEG-NF. This effect occurred together with a larger cerebral blood oxygenation slope in frontal areas. Overall, the mentioned studies demonstrate the feasibility of combining VR and fNIRS technologies to recognize attentional states and modulate them through training procedures.

Recognizing attentional states can be used to monitor subject engagement in the task. In fMRI studies [23], in a state of intense focus on the performed action, the activity of areas such as the dorsolateral prefrontal cortex (DLPFC), dorsal anterior cingulate cortex (dACC), superior and inferior parietal lobe (SPL and IPL) and anterior insula (AI) was observed to increase. In contrast, the anterior medial frontal gyrus (aMFG), posterior cingulate cortex (PCC), and some parts of the lateral parietal cortex (LPC) are more active during mind wandering, attention drifts, or relaxation. The first group of structures is referred to as a “task-positive network” and the second as a “task-negative network” due to their association with involvement in the performed activity. As demonstrated by Harrivel et al. [23], it is possible to effectively recognize the activity of these two brain networks based on the signal from DLFPC and MFG areas recorded using fNIRS.

An important aspect of evaluating the usefulness of neurotechnology is the user′s subjective experience of the interaction with the system. In conclusion, there have been many studies focused on the efficiency of classifying brain states, which omit the user′s attitude towards the use of the system. Therefore, user-centered design (UCD) in evaluating the usability of BCI and other neurotechnologies has been postulated [23]. In this model, one of the assessable dimensions is satisfaction measured by visual scales and questionnaires [24]. The same factor is also a subcomponent of usability in the Usability, afFEct, Ergonomics, and quality of Life (uFEEL) framework for User-BCI System measurements [25].

This paper aims to evaluate the applicability of a mobile CW-fNIRS system to the recognition of task-positive/task-negative network activity during the performance of the n-back task in the immersive VR environment. We hypothesize that discrimination between the enhanced attention engagement (n-back task) and the relaxation state based on hemodynamic activity (HbO/HbR concentration changes) from the DLFPC and MFG areas will be significantly higher than the level-of-chance threshold in the study group. We have used the method described in the paper by Müller-Putz et al. [26] as a measure to determine the random threshold, which considers the number of trials and categories in analyzed datasets. Furthermore, we assume that the user experience of interaction with the system (fNIRS + HMD) will be rated above the mean values for the satisfaction assessment scales. Confirmation of the hypotheses would provide evidence for the feasibility of using mobile CW-fNIRS systems to detect and train attentional states in an attractive VR environment.

## 2. Materials and Methods

### 2.1. Participants

Twelve right-handed subjects (10 females) aged 21–34 years (M = 24.82; SD = 4.38) participated in the experiment. Written informed consent was obtained from all participants in the experiment. They also declared that they were neither permanently taking medication nor taking psychoactive substances. At the end of the experimental procedure, participants were paid a remuneration of 100 PLN. The study was conducted in compliance with the Declaration of Helsinki and approved by the Ethics Committee of the Institute of Psychology at the John Paul II Catholic University of Lublin (No. KEBN_48/2021).

### 2.2. Apparatus

**Figure 1:**
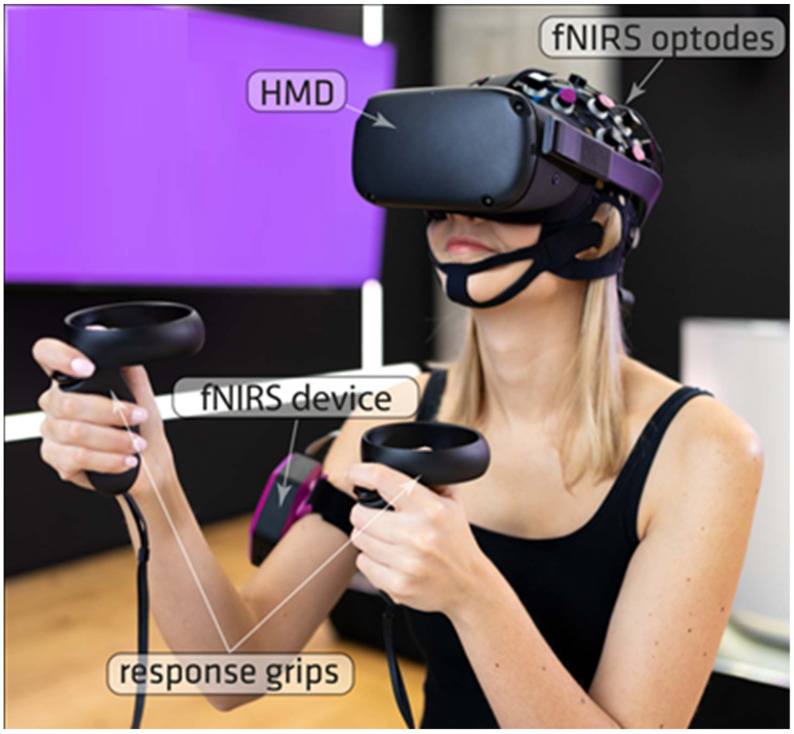
The experimental setup included wireless all-in-one VR goggles and a wearable fNIRS device. Participants were seated during data recording and the devices were not connected by a cable.

Optical signals were recorded on a two-wavelength (760 and 850 nm) continuous-wave Photon Cap C20 system (Cortivision sp. z o.o., Lublin, Poland) with 16 LED sources and 10 SiPD detectors. Data processing in online mode was conducted in OpenViBE 3.1.0 (Inria Hybrid Team, Rennes, France) with custom Python scripts. VR scenarios were developed using the Unity3D engine and displayed on an all-in-one HMD Oculus Quest (Facebook Technologies, Menlo Park, CA, USA) (Figure 1). Statistical analysis of the results was conducted using JASP v0.15 (JASP Team) software.

### 2.3. Subjective Satisfaction Assessment

An electronic version of the Visual Analogue Scale (VAS) [27] was used to assess overall subjective satisfaction after both sessions (Question: “What is your overall satisfaction with participating in this experiment?”). The evaluation was performed using an 11-point scale, where 0 means “not satisfied at all” and 10 means “very satisfied”. Then, a modified version of the Quebec User Evaluation of Satisfaction with assistive Technology (eQUEST 2.0) [28] was used to measure participants′ satisfaction regarding the seven aspects of using the system: Dimensions, Weight, Adjustment, Safety, Reliability, Ease of use and Comfort (Question: ‘’How would you rate the features of the device you came in contact with? Dimensions/Weight/Adjustment/Safety, Reliability/Ease of use/Comfort”). Subjects responded by choosing one of five possible answers by assessing each aspect: 1 = not satisfied at all, 2 = not very satisfied, 3 = more or less satisfied, 4 = quite satisfied and 5 = very satisfied.

### 2.4. Procedure

**Figure 2:**
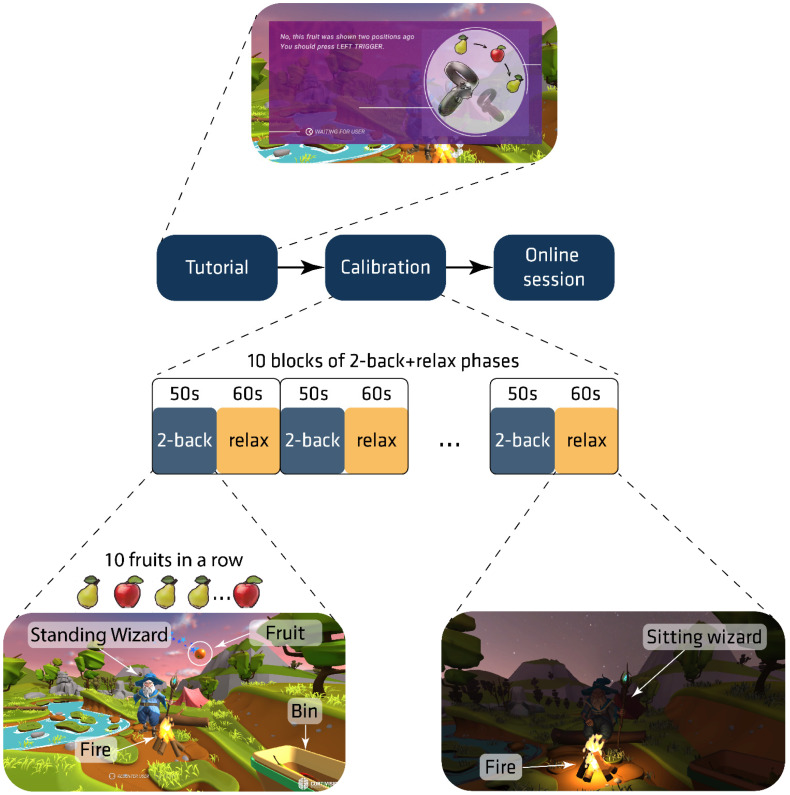
Experimental procedure. The upper screenshot shows a sample message with instructions on how to complete the task correctly (**top central**). The bottom screenshots present the virtual scene view during the 2-back block (**bottom left**) and relax (**bottom right**).

The study consisted of three parts (Figure 2). In the first stage (tutorial), written instructions were given: “You will see different fruits in this game. Each fruit is shown for a few seconds. You need to decide whether you saw the same fruit two fruits ago. If you saw the same fruit two fruits ago, you press the LEFT TRIGGER. If not, you press the RIGHT TRIGGER. If you do this correctly, the fruit will fall into the basket. The fruit will fall into the fire if you press the wrong trigger. Occasionally, the Wizard will throw a ball of energy instead of fruit in your direction. Then it will get darker and your task will be to relax and look at the fire”. Then, ten training trials with information on correctness were presented to the participants. If they felt confident enough to perform the task, a second part (calibration) began. The calibration session had the same elements as the tutorial (10 objects in a row) except for the information on correctness. In the last part (online), participants were given the following instructions: “Do the same task, but only in your mind. Try to get all the fruits into the basket. If you focus and relax at the right time, the fruit will fall into the basket”. During the online session, the movement direction of objects (“basket” or “fire”) was directly controlled by the classification output signal. In all parts of the experiment, participants saw a virtual Wizard character in front of them. In the “2-back” blocks, the character threw fruit toward the participant, while the character remained at rest in the “relax” block. Each part of the study started and ended with the “relax” block, with conditions alternating sequentially. During each “2-back” block, a list of fruits was randomized with 3 targets, 7 non-targets, and no lures.

### 2.5. fNIRS Data Acquisition and Analysis

#### 2.5.1. Probe Design

**Figure 3:**
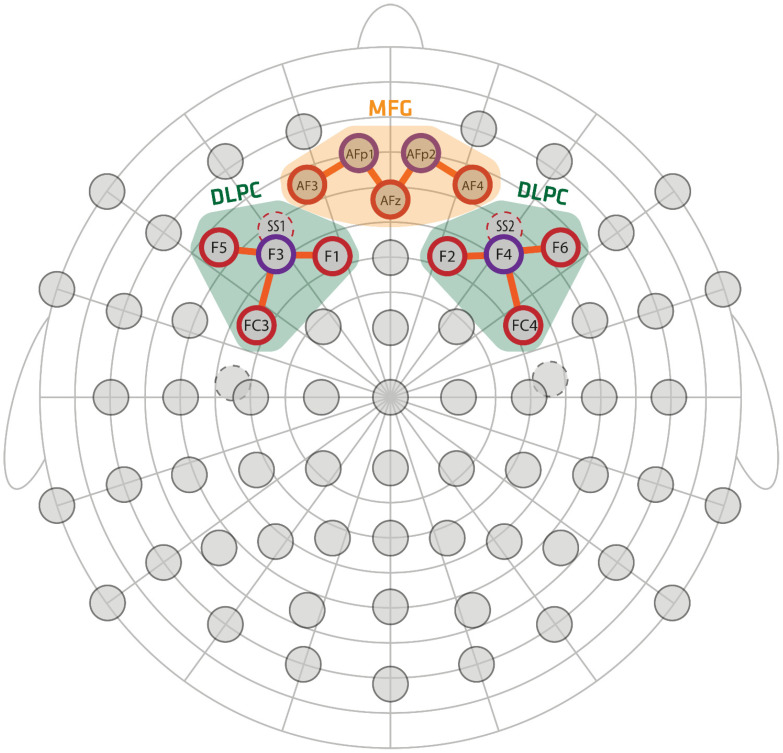
Optode montage. The source′s positions are red, and the detectors are purple. The colored fields indicate the regions of interest: green (DLPFC) and orange (MFG). The orange lines indicate the location of the fNIRS channels.

Ten channels were composed of 9 sources and 4 detectors, covering MFG and bilateral DLPFC. In addition, two short-separation channels were placed in positions F3 and F4 (see Figure 3). The distance between sources and detectors was maintained at approximately 30 mm for all data channels and was fixed to 10 mm for short-separation channels. The optode placement choice was based on predefined positions implemented in the fOLD toolbox [29]. Three regions of interest (MFG, L-DLPFC, R-DLPFC) were automatically translated from anatomical landmarks to 10-5 international system positions.

#### 2.5.2. Signal Processing

**Figure 4:**
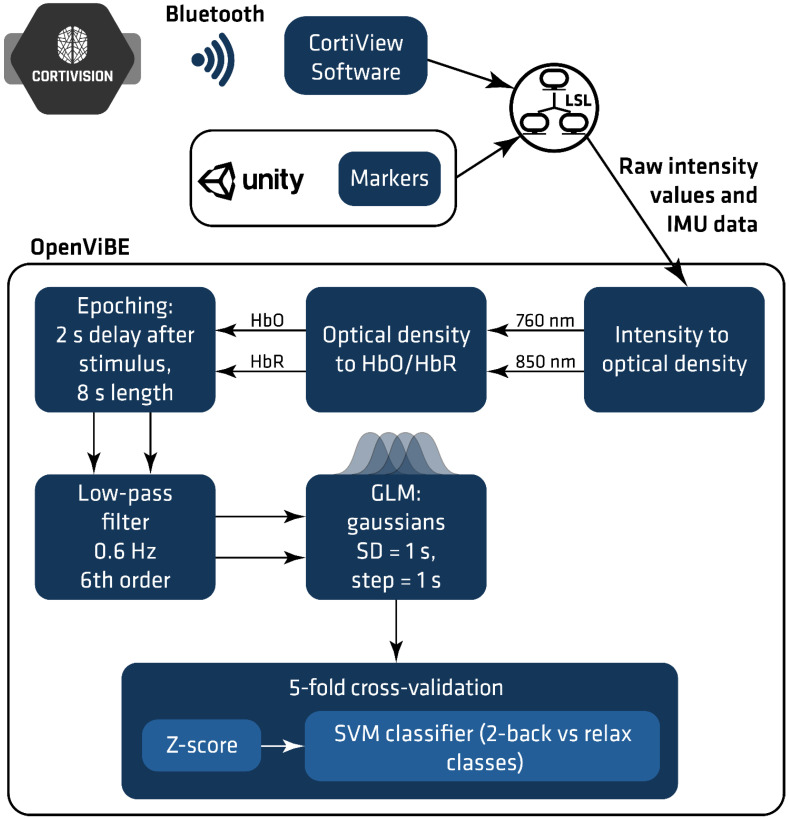
Signal processing pipeline in OpenViBE environment for data from calibration and online session.

The signal processing pipeline included several steps (Figure 4). In the first stage, the raw light intensity was transformed to optical density via the application of the modified Beer–Lambert Law [3]. The baseline necessary for calculating optical density was registered during the course of the first 5 s of the processed record. Optical density was then transformed to the concentration of oxy-/deoxy-hemoglobin (HbO/HbR) and the values for HbO and HbR concentrations were later processed separately. Stimulation-based epoching was carried out afterward, which was based on the markers streamed from the VR applications. Each epoch was 8 s long and began 2 s after the respective marker appeared. As temporal filtering, a 6th order Butterworth Low Pass Filter with a high cut frequency equalling 0.6 Hz was applied.

A generalized linear model (GLM) block was applied as a feature extraction step. A sequence of Gaussian “bell”-shaped functions was used as the model function in GLM. The standard deviation of the function was equal to 1 and the subsequent bell curves were shifted by 1 s. The GLM block returns two signals: the values of the regressed signal reconstructed from model functions and the parameters of the fit model functions (heights of the bell curves). The latter were used as features in the further analysis [30].

The obtained dataset containing extracted features was divided into five parts to conduct a 5-fold cross-validation [31] experiment, in which each fold was used as a test set, while the remaining four folds were used as a training set. Each repeat of cross-validation included the following steps:

1. *z-score* normalization: the mean and standard deviation of separate features were computed based on the training set; the values were used for z-score normalization of the training and test datasets.
2. SVM classifier [32] has been used to distinguish between two classes: “relax” and “2-back task”. The linear kernel was applied as this is less prone to overfitting than other kernels.

## 3. Results

### 3.1. Classification Accuracy

**Figure 5:**
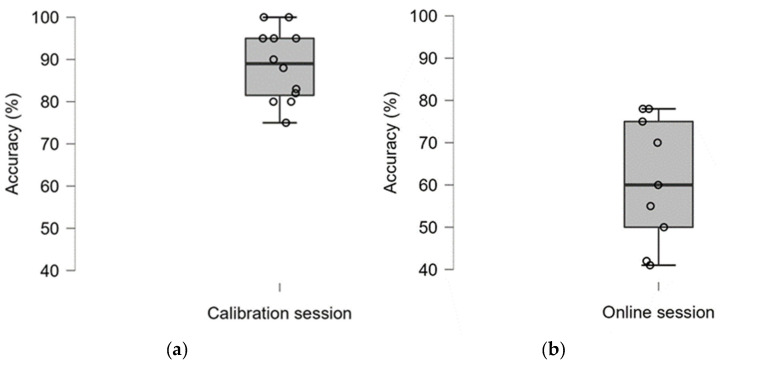
Classification accuracy of calibration (**a**) and online sessions (**b**). The boxplots show the median and interquartile range, whiskers indicate the range.

The results of the one-sample non-parametric Wilcoxon signed-rank test show that classification accuracy in the calibration session (*M* = 88.58, *SD* = 8.49) is significantly higher than the upper 95% confidence limit of chance calculated for two-class BCI according to Müller-Putz et al. [26] (70%) (*T* = 78, *p <* 0.01, *r_rb_* = 1). However, we did not note a significant difference for online conditions (*M* = 61, *SD* = 14.89, *T* = 6, *p* = 0.107). At the same time, both conditions were significantly higher from the 50% chance level (calibration: *T* = 78, *p <* 0.001, *r_rb_* = 1; online: *T* = 31, *p <* 0.05, *r_rb_* = 0.72). Table 1 presents the results of the mean classification accuracy of all participants in calibration and online sessions. It has to be noted that three results are missing from the online session due to errors during signal recording. Figure 5 shows the distribution of outcomes in both parts of the study, referred to as the level of chance.

### 3.2. User Satisfaction

Users′ overall satisfaction with their interaction with the system averaged 6 sten, which was close to the maximum rating of “very satisfied” on an 11-point scale (Table 2. VAS). The highest-rated aspects of the interaction were found to be Safety and Ease of use. An average rating above five sten was assigned to the remaining aspects of the system: Adjustment, Dimensions, Reliability, Weight, and Comfort (Table 2. eQUEST 2.0).

## 4. Discussion

According to the hypothesis, the classification results in the calibration session confirmed the possibility of discrimination between the enhanced attention engagement (n-back task) and the relaxation state based on HbO_2_/HbR concentration changes from the DLFPC and MFG area. The classification accuracy at an average level of 88.58% is significantly above the confidence intervals (α = 5% and α = 1%) of the random effect for a two-category procedure containing 20 trials [26]. These outcomes are higher than those reported in other studies using fNIRS to identify cognitive engagement/relaxation based on task-positive/task-negative network activities (*M* = 69.1%; *SD* = 4.4%) [23]. Slightly lower recognition effect between the 2-back and relax condition (*M* = 80.3%; *SD* = 10.45%) was also achieved by analyzing average activity from eight channels located over the prefrontal cortex without distinguishing between the DLFPC and MFG area [33]. However, such an effect was not observed for the data collected in the online session (*M* = 61, *SD* = 14.89). The mean classification outcomes from the online session are significantly above the chance threshold (50%) but below the upper confidence interval for a more conservative measure proposed by Müller-Putz et al. [26]. Although such a result remains relatively low, an average classification accuracy of more than 60% is sometimes considered the minimum correctness threshold for most users of 2-state BCI after training [34].

The classification results can be interpreted in the context of participants′ tasks in both parts of the study. In the calibration phase, subjects had to respond to stimuli in the VR environment by pressing the correct trigger; in contrast, in the online phase, the stimuli control was based only on the classifier′s output training using the data from the calibration phase. This implies two types of problems. First, subjects may have been more focused on the task during the offline sessions due to the need for motor responses. During online control, participants′ attention may have drifted or they may have felt a lack of control over the procedure, which is a common problem in BCI research [35]. Secondly, for the same reasons, the calibration session data used to train the classifier in the online session may not have been representative of the activity in this phase.

The satisfaction evaluation methods show overall positive user enjoyment when interacting with the system. These results are consistent with research indicating that virtual environments increase user engagement during cognitive tasks [21]. Head-mounted displays are still a relatively uncommon entertainment device, meaning that contact with this technology is likely to elicit user interest [36]. However, overall satisfaction scores may have been influenced by positive skew bias. It has been observed that subjects tend to choose positive scores when answering questions on satisfaction [37]. Indeed, in our study, all participants only selected answers from among the highest scores (from 8 to 10 on an 11-point scale). Therefore, it may be more valuable to assess satisfaction with specific technology features as rated by the eQUEST scale.

Concerning the eQUEST results, HMD and fNIRS are viewed by users as safe and easy to use. This may be due to the small sizes (all-in-one HMD and wireless fNIRS) and short setup time for both devices. However, aspects directly related to physical convenience during the interaction, such as Weight, Comfort, and Adjustment, were rated ambiguously. The weight of both devices on the head is nearly a kilogram (HMD = 503 g; fNIRS optodes and cap = 265 g), which can be uncomfortable during a more extended session. In addition, the EEG cap system (MCScap 10-5, Medical Computer System, Sankt-Petersburg, Russia) used to attach the fNIRS optodes is designed for a relatively wide range of head circumferences (e.g., 54 cm to 60 cm for size L). Thus, the cap may have appeared unsuitable for some participants at the beginning or end of the size range. In summary, it can be assumed that the subjects are positively disposed towards the technology that composed the HMD-fNIRS system. The method appears safe and easy to use. However, user comfort can be improved.

There are some limitations of our study that need to be mentioned. First, the small number of subjects and trials has made it difficult to use more advanced machine learning algorithms, such as deep neural networks [38]. Therefore, the classification results should not be generalized to other cases without replicating the effect on larger data sets using different methods. Another limitation is the lack of comparisons to other conditions, such as performing cognitive tasks presented on a monitor screen. Such comparisons would allow us to verify whether the use of HMDs affects the accuracy of task-positive/negative brain network activity recognition. It would also be beneficial for future research to include more user experience factors than satisfaction [24,25].

## 5. Conclusions

As hypothesized, the classification of hemodynamic changes from the areas of interest allowed the recognition of states of enhanced attention and relaxation above a level of chance threshold. HMD and mobile fNIRS in the experiment resulted in high overall user satisfaction with system interaction. Moreover, the signal processing pipeline in open-source real-time data processing software can provide a framework for future BCI and neurofeedback studies. For example, research [39] shows that it is possible to achieve symptom improvement in social anxiety disorder (SAD) by attention-focused neurofeedback training using the VR-BCI fNIRS setup. The same techniques can create more realistic social stimuli or interactions to evoke and recognize emotional states [40]. A further area to apply the VR-fNIRS combinations might also be the study of memorizing and reproducing processes [41]. In summary, due to the mobile capabilities of modern fNIRS and HMD systems, it is possible to study cognitive processes in more realistic but controlled conditions.

## Figures and Tables

**Table 1:** Results of classification for individual participants (*n* = 12).

Subject	Calibration Session	Online Session
A	80	50 *
B	88	-
C	83	42 *
D	82 *	70
E	95	75
F	95	41 *
G	100	78
H	75	60 *
I	95	-
J	90	55 *
K	80	-
L	100	78
Group	*M* = 88.58; *SD* = 8.49	*M* = 61; *SD* = 14.89

* Results below 95% confidence limits of chance.

**Table 2:** Satisfaction assessment during the session (*n* = 11, missing case = 1).

Method	Dimension	Min	Max	Md Sten	IQR Sten
VAS	Overall satisfaction	8	10	6	2.25
eQUEST 2.0	Dimensions	2	5	6	4
	Weight	2	5	6	2
	Adjustment	2	5	7	3.5
	Safety	4	5	10	0
	Reliability	4	5	6	4
	Ease of use	3	5	10	5
	Comfort	1	5	6	1
VAS (0 = not satisfied at all to 10 = very satisfied) eQUEST 2.0 (1 = not satisfied at all, 2 = not very satisfied, 3 = more or less satisfied, 4 = quite satisfied, 5 = very satisfied).				Median (Md)	Interquartile range (IQR)

## Data Availability

The data presented in this study are available on request from the corresponding author. The data are not publicly available due to participants’ lack of written consent to publish their data in an open repository.
